# Anisotropic structural and optical properties of semi-polar (11–22) GaN grown on *m*-plane sapphire using double AlN buffer layers

**DOI:** 10.1038/srep20787

**Published:** 2016-02-10

**Authors:** Guijuan Zhao, Lianshan Wang, Shaoyan Yang, Huijie Li, Hongyuan Wei, Dongyue Han, Zhanguo Wang

**Affiliations:** 1 Key Laboratory of Semiconductor Materials Science, and Beijing Key Laboratory of Low Dimensional Semiconductor Materials and Devices, Institute of Semiconductors, Chinese Academy of Sciences, P.O. Box 912, Beijing 100083, People’s Republic of China

## Abstract

We report the anisotropic structural and optical properties of semi-polar (11–22) GaN grown on *m*-plane sapphire using a three-step growth method which consisted of a low temperature AlN buffer layer, followed by a high temperature AlN buffer layer and GaN growth. By introducing double AlN buffer layers, we substantially improve the crystal and optical qualities of semi-polar (11–22) GaN, and significantly reduce the density of stacking faults and dislocations. The high resolution x-ray diffraction measurement revealed that the in-plane anisotropic structural characteristics of GaN layer are azimuthal dependent. Transmission electron microscopy analysis showed that the majority of dislocations in the GaN epitaxial layer grown on m-sapphire are the mixed-type and the orientation of GaN layer was rotated 58.4° against the substrate. The room temperature photoluminescence (PL) spectra showed the PL intensity and wavelength have polarization dependence along parallel and perpendicular to the [1–100] axis (polarization degrees ~ 0.63). The realization of a high polarization semi-polar GaN would be useful to achieve III-nitride based lighting emission device for displays and backlighting.

III-nitride semiconductors have been widely used for the light emitting devices (LEDs) in the green, blue and ultraviolet (UV) spectral range^1^. The commercially available GaN-based devices are mostly grown on *c*-plane sapphire. However, the III-nitride epilayers grown along the c-axis ([0001]) of the wurtzite crystal structure suffer from spontaneous and piezoelectric polarization fields which induce the huge quantum-confined Stark effect (QCSE). From the point of view of device performance, the presence of these electric fields is undesirable since they considerably reduce the emission efficiency of GaN-based LEDs^2^. Therefore, it is especially important for high efficient LEDs to circumvent the effect of internal electric field. Several groups recently proposed the growth of non-polar and semi-polar nitrides as a mean to reduce the magnitude of the internal polarization-induced electric fields along the growth direction^2^,^3^,^4^,^5^,^6^. For examples, non-polar GaN layers were grown on the *r*-plane^7^ and patterned *a*-plane sapphire^8^, or semi-polar GaN layers on *m*-plane sapphire^9^, and non-polar *m*-plane (1–100) InGaN LEDs on the *m*-plane (1–100) GaN substrates^4^, as well as semi-polar (11–22) InGaN LEDs on semi-polar (11–22) GaN bulk substrates^10^, etc. The semi-polar orientation such as (11–22) plane is especially promising due to the high In incorporation into InGaN active layers^10^,^11^ and the suppression of the quantum-confined Stark effect (QCSE) in quantum-well (QW) structures^12^,^13^. However, experimental studies of these (11–22)-oriented QW devices, suffer from limitations in GaN bulk substrate supply and high-cost, whereas semi-polar (11–22) GaN films grown on *m*-plane sapphire have still suffered from poor crystal qualities and rough surfaces because of anisotropic crystallographic mismatch between semi-polar (11–22) GaN and m-sapphire^9^,^14^,^15^. To improve the crystallite quality of semi-polar GaN crystal quality, a few approaches for MOCVD growth of (11–22) GaN have been reported^16^,^17^,^18^,^19^. For example, nitridation and Si/N treatment^16^, without low-temperature GaN buffer layer^17^, epitaxial lateral overgrowth (ELO)^18^ and controlling niridation condition^19^. Another challenge in heteroepitaxy of (11–22) III-nitrides on m-plane sapphire is (10-1-3) mixed phases. To reduce the contributions of unwanted (10-1-3)-oriented phases, a few methods for MOCVD growth of (11–22) III-nitrides have also been reported^20^,^21^,^22^,^23^. The common procedure of these methods is nucleation of AlN or AlN buffer layer. The aims of all the different approaches are phase purity, i.e. maximize the crystal quality as indicated by the FWHM of X-ray rocking curves and obtain smooth surfaces. Unlike the polar (0001) plane, non-polar and semi-polar planes have low crystal symmetries, which cause the semi-polar and non-polar GaN and InGaN/GaN quantum wells (QWs) exhibit optical gain anisotropy and thus emit polarized light^24^. It has been suggested that this phenomenon is related to the anisotropic strain in the GaN film and QWs^25^. The optical anisotropy directly reflects the energy-band structures of emitting layers. However, the spectroscopic investigation on semi-polar (11–22) GaN has rarely been reported in spite of the significant interest on the growth of semi-polar (11–22) GaN on different substrates by various techniques. In this work, we present a three-step growth approach with double AlN buffer layers to substantially improve the quality and surface morphology of (11–22) GaN, which is evaluated by x-ray diffractometric, transmission electron microscopic (TEM), scanning electron microscopic (SEM), and atomic force microscopic (AFM) measurement. The polarization anisotropy of (11–22) GaN was studied by photoluminescence (PL) spectroscopy.

## Results and Discussion

**Figure 1:**
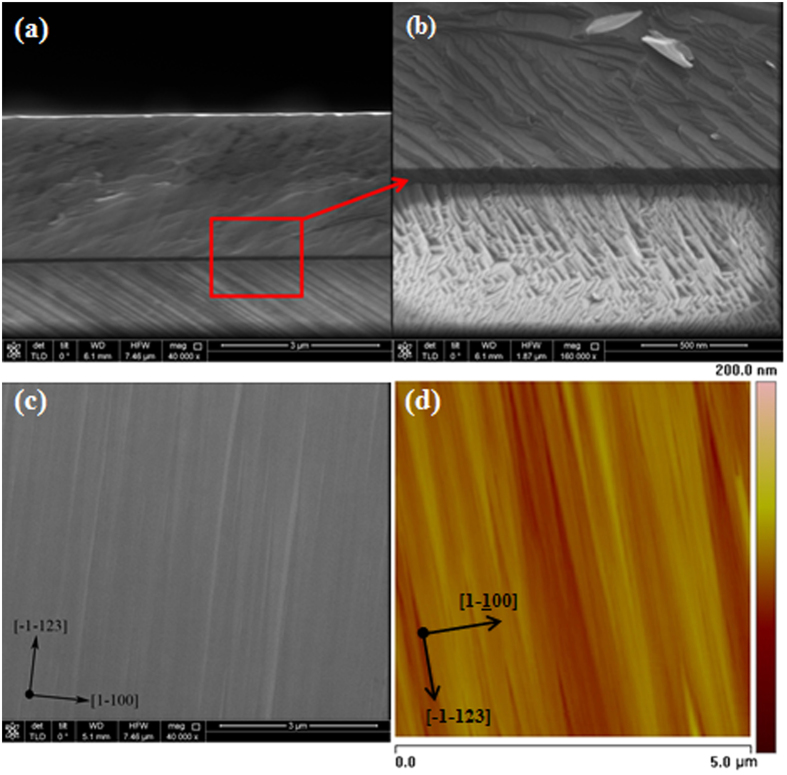
(**a**) A Cross-sectional SEM image of the (11–22) GaN layer grown on the *m*-plane sapphire. (**b**) Enlarged SEM view of the AlN buffer layer. (**c**) A plane-view SEM image for the (11–22) GaN layer. (**d**) An AFM image of the GaN surface. The sample shows arrowhead-like features along the [–1–123] direction. The root mean square (RMS) roughness of the GaN layer is 9 nm.

The cross-sectional SEM images of the semi-polar (11–22) GaN layers grown for 2 hours at 1050 °C are shown in Fig. 1(a,b). Apparently, the 2.5 μm-thick semi-polar (11–22) GaN layer which grown on m-sapphire with an 80 nm AlN buffer layer exhibits perfect morphology. Figure 1(c) shows the surface morphology of the GaN layer measured by SEM. There are a few arrowhead-like facets on the surface. This kind of surface morphology is believed to originate from the anisotropic lattice mismatch between semi-polar (11–22) GaN and m-sapphire^26^. A typical AFM image of 2.5 μm-thick semi-polar (11–22) GaN grown on *m*-plane sapphire is shown in Fig. 1(d). Similar to the observation by SEM, the surface of (11–22) GaN layer exhibits “facet-like” features with a root mean square (RMS) roughness below 9 nm over 5 × 5 μm^2^ surface area, which is comparable to previous reports^23^,^27^,^28^. The typical arrowhead-like surface morphology of semi-polar (11–22) GaN film was developed by using the heteroepitaxial growth. We speculated that the anisotropic arrowhead-like surface structure may be caused by the crystallographic difference between *m*-plane sapphire and semi-polar (11–22) GaN due to the different incorporation probability and diffusion length of surface atoms along different crystallographic directions.

**Figure 2:**
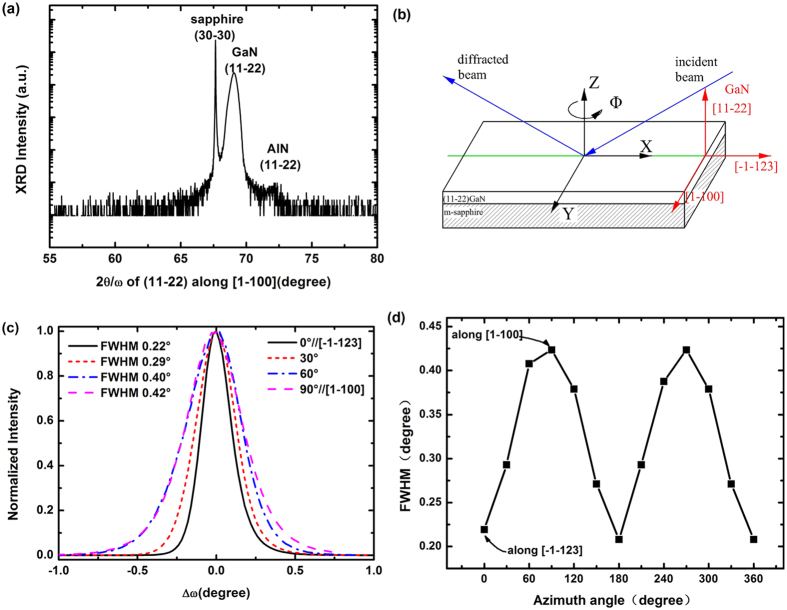
(**a**) 2θ-ω scan profile of the symmetric plane of the GaN layer along [1–100]. (**b**) Experimental geometry of the azimuthal dependent XRD measurement, defining the azimuth angle as zero when the projection of the incident beam is parallel to the [-1-123] GaN axis.(**c**) XRD *ω* rocking curves of (11–22) GaN reflection in four azimuth angles. (**d**) Azimuth angle dependence of the FWHM of the (11–22) *ω* rocking curves.

To clarify the orientation of the GaN layer, XRD analysis was performed. Figure 2(a) shows a 2*θ-ω* scan profile from the symmetric (11–22) plane of the GaN layer while the X-ray incident beam is parallel to the [1–100] GaN direction. Only a (11–22) diffraction peak from GaN, a (11–22) diffraction peak from AlN and a (30–30) diffraction peak from sapphire were observed. Thus, a singular oriented {11–22} GaN layer was obtained, indicating growth of a pure {11–22} GaN layer on a foreign substrate. Using three-step growth method which consisted of a low temperature AlN buffer layer followed by a high temperature AlN buffer layer, the contributions of unwanted (10-1-3)-oriented phases^28^ is almost non-existent. The pure (11–22) GaN has been attained.

To reveal the anisotropic characteristics of a typical semi-polar GaN layer, the ω-rocking curves of the (11–22) reflection of GaN layer were measured as a function of the azimuth angle *Φ*, as schematically shown in Fig. 2(b). The azimuth angle was defined as zero degree when the projection of the incident beam was parallel to the [-1-123] GaN direction. Figure 2(c) shows the XRD ω scanning of the (11–22) refection of GaN layer at azimuth 0°, 30°, 60° and 90°. Similar to *a*-plane GaN on *r-*plane sapphires^25^, the X-ray rocking curve (XRC) of the symmetric (11–22) GaN reflection is anisotropically broadened about 0.22° FWHM along [-1-123] GaN direction and 0.42° FWHM along [1–100] GaN direction. Here, we compared our GaN sample (with 20 nm LT-AlN and 60 nm HT-AlN buffer layers) with Sun *et al.*’s sample A^22^, which only used 67 nm HT-AlN buffer layer. The FWHMs were reported to be 0.4° at *Φ* = 0° and 0.63° at *Φ* = 90°, indicating that the two AlN buffer layers could improve the structural quality of (11–22) GaN. An M-shaped azimuthal dependence of FWHM values over 360° angle range was observed in Fig. 2(d), which is similar to the observation reported for MOCVD a-GaN^29^ and (11–22) GaN^22^,^30^,^31^,^32^,^33^. The broadening of (11–22) XRC FWHM along the [1–100] direction is caused by the larger distortion of the GaN lattice due to higher density of defects such as threading dislocations, stacking faults (SFs), as well as a larger mosaic tilt and/or a reduced coherent length (smaller size of the mosaic blocks)^32^,^34^.

**Figure 3:**
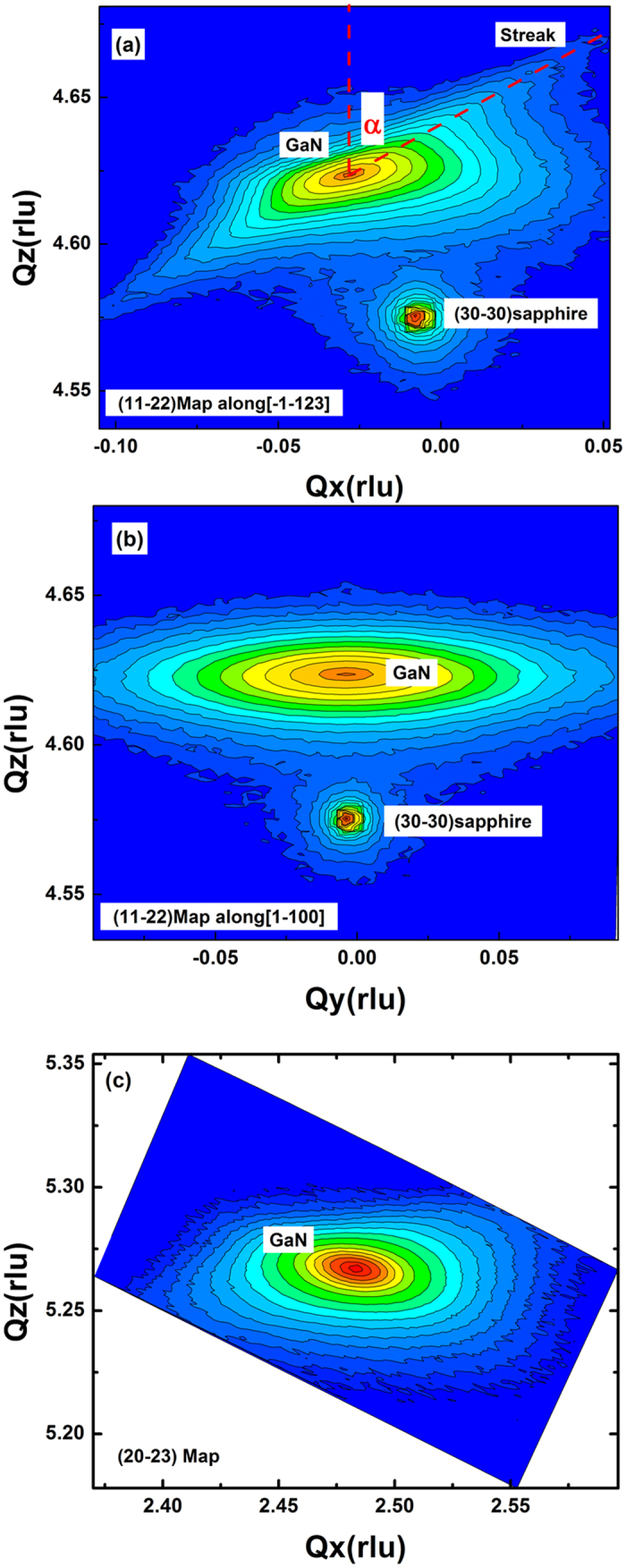
X-ray reciprocal space maps of the semi-polar sample near the symmetric (11–22) reflections of GaN and the symmetric (30–30) reflection of *m*-plane sapphire, measured at two different in-plane directions [−1–123] (**a**) and [1–100] (**b**). (**c**) X-ray reciprocal space maps of (20–23) reflections.

To completely understand the XRD broadening mechanism in the semi-polar (11–22) GaN, the XRD reciprocal space maps (RSM) for the (11–22) symmetric reflection and the (20–23) asymmetric reflection were taken (Fig. 3). Figure 3(a,b) correspond to the reciprocal space mapping of the semi-polar GaN layer measured at two different azimuth angles, in a configuration where the scattering plane is either parallel to the [-1-123] direction or parallel to the [1–100] direction. These spots indicate that, under our MOCVD growth conditions, the surface of GaN on *m*-plane sapphire is (11–22) plane. The maximum peak intensity for the (11–22) reflection along [-1-123] direction has the components Q_x_ = −0.027, Q_z_ = 4.624 Å^−1^, while the maximum peak intensity for the (11–22) reflection along [1–100] direction has the components Q_x_ = −0.004, Q_z_ = 4.624 Å^−1^. The maximum peak intensity of the (20–23) reflection is located at the scattering wavevector **Q**(20–23) = (Q_x_ = 2.483, Q_z_ = 5.267) (Å^−1^). All diffraction spots are aligned along the abscissa, indicating that GaN and Al_2_O_3_ surface planes are exactly parallel to each other. As pointed out by the results in a literature^35^,^36^, the reciprocal lattice points (RLPs) will be elongated along and parallel to the Q_x_ axis if the broadening is caused dominantly by the limited mosaic block dimensions. The RLPs should tilt in the reciprocal space if an additional mosaic tilt exists in the sample. As shown in Fig. 3(a,b), the (11–22) RLPs along [-1-123] and [1–100] of the GaN sample are all predominantly broadened in the Q_x_ direction, indicating that the dominant broadening mechanism for it is the limited mosaic block size. In the Fig. 3(a), the inclination α(α~58.4°) of the sharp streak with respect to the Q_z_ axis will be taken as measure for the RLP orientation, indicating that the streak parallel to the direction [0001]. This streak is caused by diffuse scattering from the basal plane stacking faults (BSFs). The intensity is clearly correlated with the BSFs density, i.e., the higher diffuse scattering, the more BSFs^23^,^36^.

**Figure 4:**
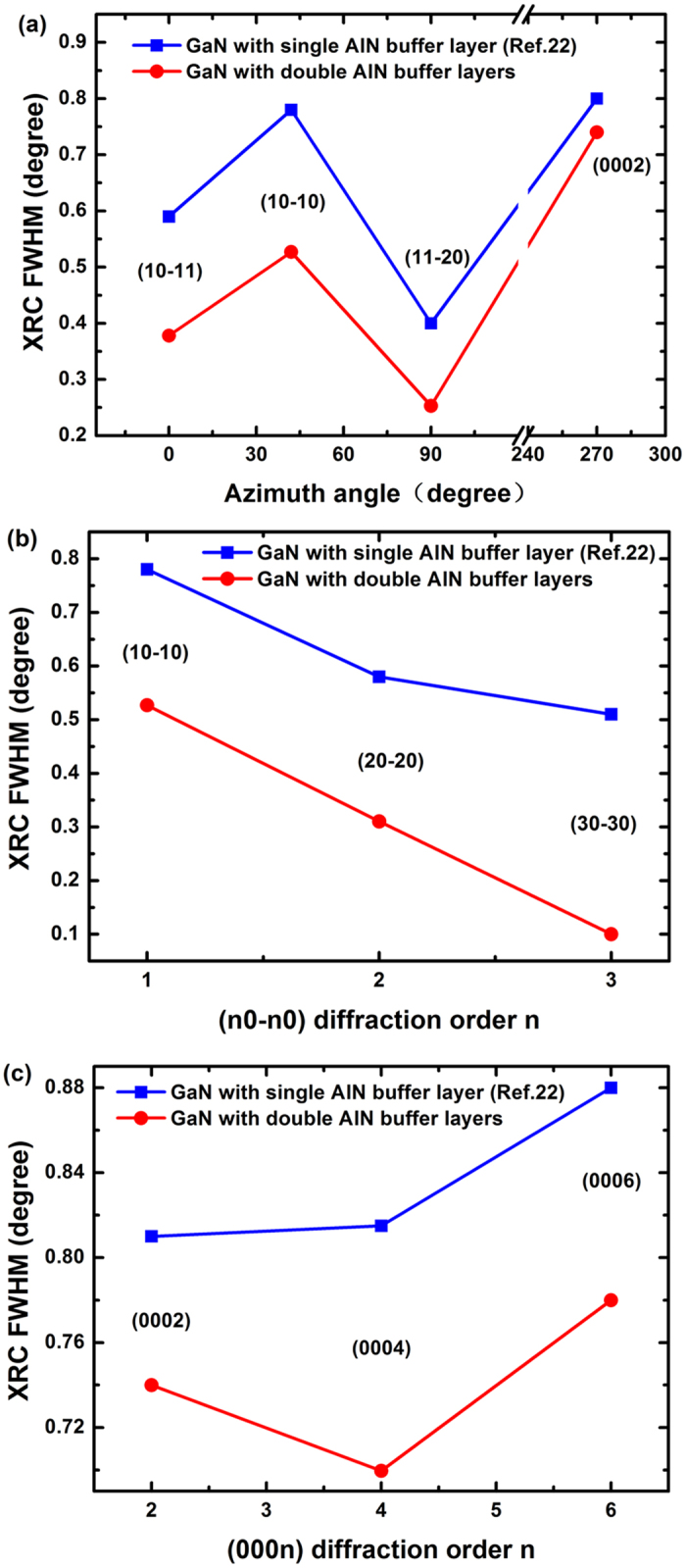
(**a**) The FWHM of the off-axis XRC of (11–22) GaN as a function of the azimuthal angle, *Φ*. (**b**) The FWHMs of m-plane (*n*0-n0) XRCs (*n* = 1, 2, and 3). (**c**) The FWHMs of c-plane (000*n*) XRCs (*n* = 2, 4, and 6).

Figure 4 shows the FWHM of XRCs in a *skew* symmetric geometry for off-axes diffraction planes with an inclination angle *Φ* with respect to (11–22) at various azimuths (Fig. 2(b)). We compared our GaN sample (with 20 nm LT-AlN and 60 nm HT-AlN buffer layers) with Sun *et al.*^22^’s sample A (with 67 nm HT-AlN buffer layer). As shown in Fig. 4(a), we selected the following planes: (10–11), (10–10), (11–20) and (0002). The diffraction angles and origin of the broadening factors for various diffraction planes were reported by Sun *et al.*^22^. Figure 3(a) shows that with double AlN buffer layers the off-axis XRCs were narrowed down by ~30%, which evidenced that the three-step growth technique could significantly improve the microstructural quality of (11–22) GaN. In Fig. 4(b), the FWHMs of the off-axis (10–10) (20–20) and (30–30) peaks decreased by approximately 32%, 46% and 86% from 0.78°, 0.58° and 0.51° to 0.53°, 0.31° and 0.1°, respectively. On the other hand, as shown in Fig. 4(c), the FWHMs of the off-axis (000*n*) peaks decreased by approximately 10%. The XRC peaks of the various off-axis planes were found to have different broadening factors. The off-axis plane peaks of (*n*0-*n*0) were broadened by BSFs, and the FWHM of the (000*n*) peak was associated with partial dislocations (PDs) and/or perfect dislocations^22^,^37^,^38^. With double AlN buffer layers, the FWHM of XRCs for all the off-axis diffraction in Fig. 4 decreased dramatically, which indicates that the two AlN buffer layers could reduce BSFs and PDs and/or perfect dislocations.

**Figure 5:**
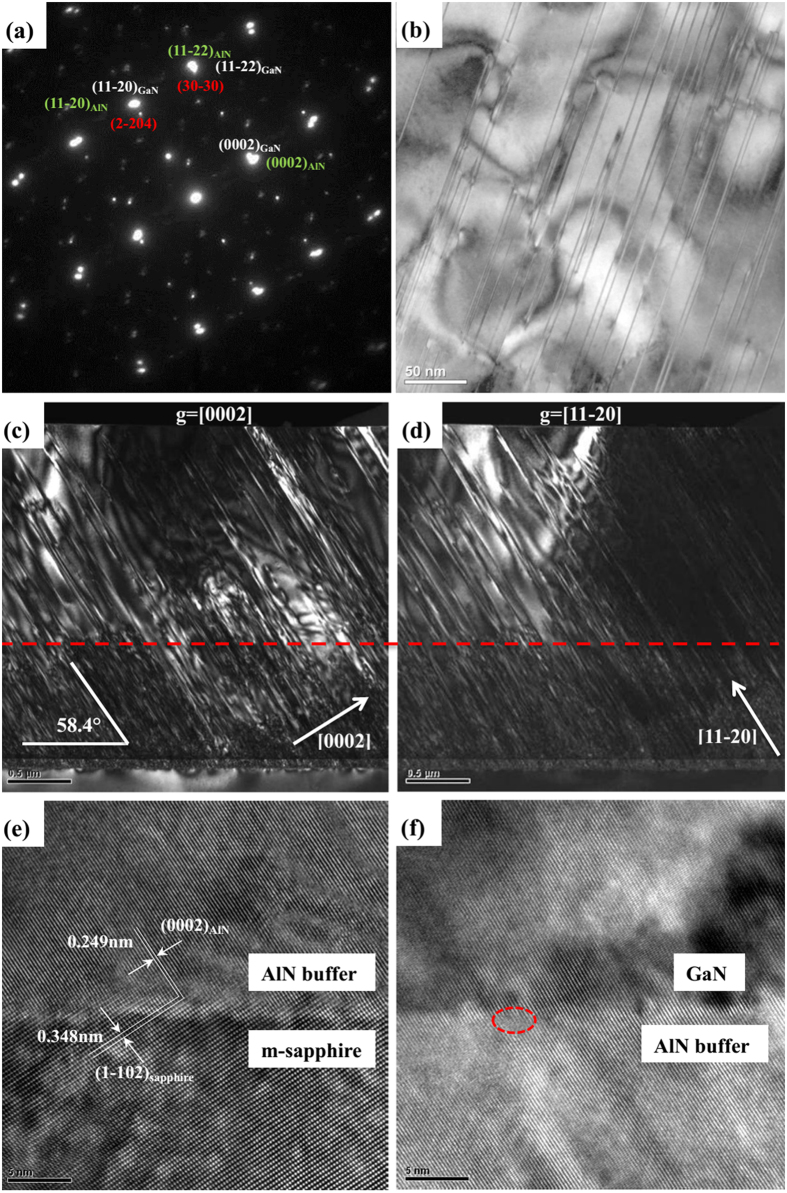
(**a**) Selected area diffraction pattern of the GaN/AlN buffer layer/sapphire region. (**b**) Plan-view TEM images of semi-polar (11–22) GaN films under two-beam conditions with **g** = 1–100. Cross sectional weak-beam dark field (WBDF) TEM of semi-polar (11–22) GaN with the reflection **g** = 0002 (**c**) and **g** = 11–20 (**d**), HRTEM images of AlN buffer/sapphire interface (**e**) and GaN/AlN buffer interface (**f**) were taken at the [10–10] zone axis, the (0002)_AlN_ and (1–102) sapphire planes are indicated by white lines.

In our GaN films, X-ray diffraction analysis has already demonstrated a {11–22} orientation. We confirm this orientation by selective area electron diffraction (SAED) analysis (Fig. 5(a)) which corresponds to the diffraction of the GaN/AlN buffer layer/sapphire region. GaN and AlN spots are the strongest ones and are indexed in white and green whereas sapphire spots are weaker and indexed in red. The <0001>_III-nitride_ direction is found inclined relative to the growth direction as expected. A small angle between the {10–10}_sapphire_ and {11–22}_AlN_ planes is observed in Fig. 5(a) (~1°). On the other hand, the {1–102}_sapphrie_ and {11–20}_AlN_ planes are found to be almost parallel. In unstrained AlN, the angle between the {11–20} and the {11–22} planes is 31.59° whereas the angle between {1102} and {1100} planes in sapphire is 32.28°. The difference is 0.69°. It appears that it is the parallelism between inclined {1–102}_sapphrie_ and {11–20}_AlN_ planes which governed the epitaxial relationships. In fact, with such an epitaxial relationship, a 0.69° angle exists between the substrate surface and the{11–22}_AlN_ direction and corresponds to the above-mentioned measured angle of ~1°. Figure 5(b) is a plan-view TEM image under two-beam conditions with **g** = [1–100]. The dark lines running across the image are BSFs. From the TEM observation, the density of BSFs was 9.6 × 10^5^ cm^−1^, similar to many previous reports^23^,^33^.

To further understand the properties of dislocations, cross-sectional TEM weak-beam dark field images were recorded with the reflection **g** = [0002] and **g** = [11–20], as shown in Fig. 5(c,d), respectively. By using extinction conditions (**b**•**g** = 0), a pure edge dislocation (Burgers vector **b** = 1/3 < 11–20 > ) can be observed only with **g** = [11–20] taken rather than with **g** = [0002], while a pure screw dislocation (Burgers vector **b** = <0001>) may be seen only with **g** = [0002]. In our case, the dislocations can be observed either with **g** = [11–20] or **g** = [0002] as shown in Fig. 5(c,d), implying that these dislocations are neither simply pure edge nor pure screw typed. They would be the mixed-type dislocation with either edge-components or screw-components. These defects have an angle of ~ 58.4° to the growth surface, and thus seem to be connected to the basal plane. Together with the observations mentioned above, the mixed-type dislocations are the majority. Large numbers of dislocations were generated at the interface and induced a highly distorted region with a thickness about 900 nm (the red dashed line in Fig. 5(c,d)). The density of dislocations above the distorted region was decreased as the layer thickness was increased. The dislocations were less observed near the top layer. This indicates that pairs of screw or edge dislocations with opposite Burgers vectors tend to annihilate each other and relax the strain of the GaN film in the subsequent deposition as more and more material is deposited. The mixed-type dislocation with multislip systems has a higher probability than the pure-edge type to annihilate dislocations by cross slip.

High-resolution transmission electron microscopy (HRTEM) was applied to gain further insight into the AlN buffer/sapphire interface (Fig. 5(e)) and GaN/AlN buffer interface (Fig. 5(f)). The interface is rather rough with the appearance of {1–102}_sapphire_ nanofacets. The (1–102)_sapphire_ and (0002)_AlN_ planes are shown by white lines in Fig. 5(e). The interplanar spacing of (0002)_AlN_ and (1–102)_sapphire_ planes is 0.249 nm and 0.348 nm, respectively. It can be assumed that AlN nucleation preferably occurred on these facets, reproducing the epitaxial relationship observed in the case of planar (1–102)_sapphire_ surfaces. With growth proceeding, the epitaxial relationships were sustained. Figure 5(f) shows cross-sectional HRTEM images of the interfacial region in the sample, revealing the atomic structure of the GaN and AlN. The red circle denotes the misfit dislocations. The misfit dislocations were not observed along the entire GaN/AlN buffer interface, possibly because they may have been obscured by contrast variations that arise as a result of variations in the orientation and thickness of the TEM specimen.

**Figure 6:**
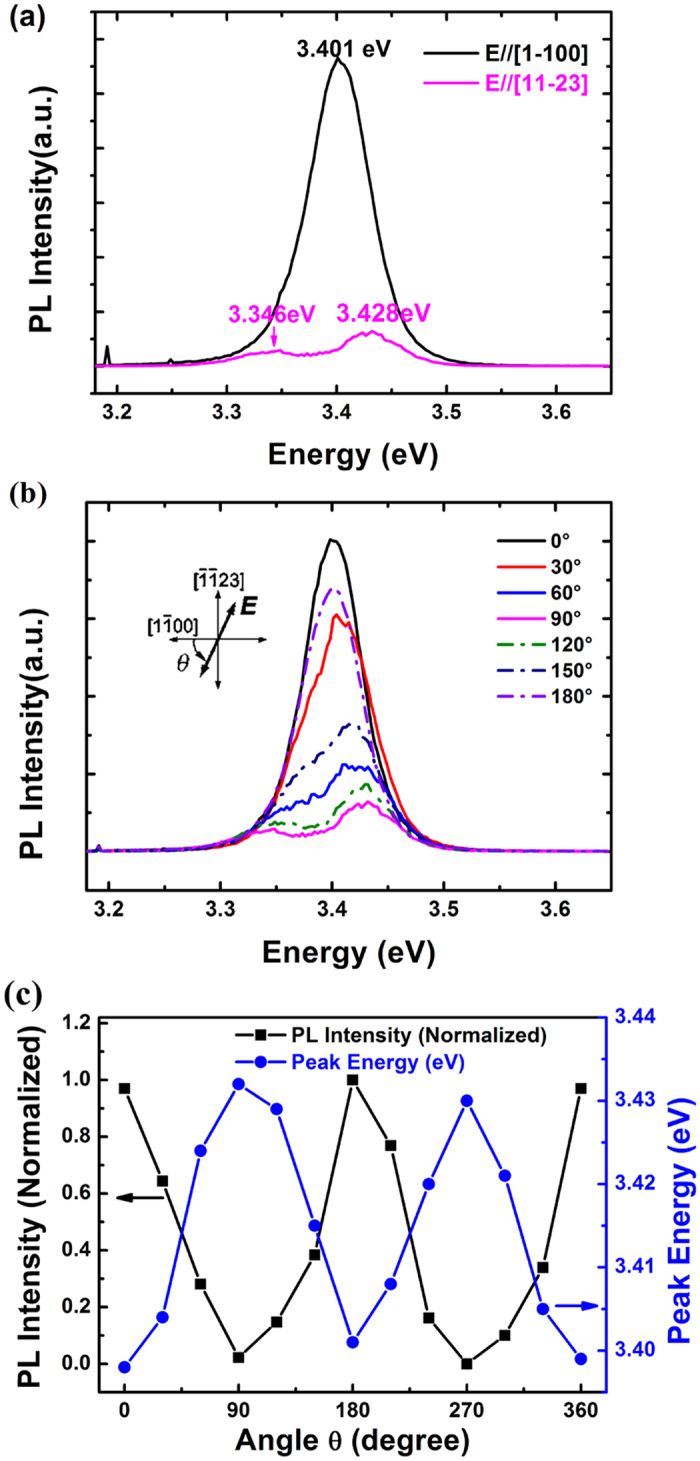
(**a**) PL spectra at polarizations parallel or perpendicular to the [1–100] axis on (11–22) GaN at room temperature. (**b**) PL spectra of (11–22) GaN at different polarization angles (θ) at room temperature. (**c**)Variation of normalized PL intensity and PL peak energy of (11–22) GaN with polarization angle *θ* at room temperature.

Due to the low crystalline symmetry of the (11–22) plane, the PL should exhibit polarization anisotropy. Figure 6(a) shows the polarized PL spectra of (11–22) GaN at 300 K, where we use a polarizer to measure the degree of polarization. The polarization-dependent PL experiment was carried out by rotating the polarization of the input laser beam with respect to the [1–100]-axis from *θ* = 0° to 180°. The *θ* *=* 0° (90°) corresponds to E|| [1–100] (E⊥[1–100]) (insert of Fig. 6(b)); where E is the electric field vector and *θ* is the polarization angle. The in-plane polarization properties were assessed using a Glan-Taylor polarizer. The signal was collected at vertical incidence. The integrated intensity is maximum for E || [1–100] and is minimum for E || [-1-123]. Furthermore, the peak position from GaN band-edge emission are shifted for the both directions, In the E || [1–100] direction, the emission peak is located at 3.401 eV, close to the peak position from *c*-GaN on *c*-plane sapphire. However, in the E || [-1-123] direction, the emission is peaked at 3.428 eV, implying the more stress in GaN layer than in [1–100] direction. The additional peak at 3.346 eV is observed, possibly from the defect-related emission. The PL spectra of our (11–22) GaN homoepitaxial layer recorded at different polarization angle *θ* at room temperature is shown in Fig. 6(b). The integrated PL intensity and the PL peak energy at different angle *θ* are shown in Fig. 6(c). It is clear that the GaN layer emits strongly polarized light. The integrated intensity is maximum for E || [1–100] and is minimum for E || [-1-123]. The polarization ratio ‘*ρ*’ is defined as: 
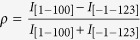
, Where I_[1-100]_ and I_[-1-123]_ are the PL intensities for E || [1–100]and E⊥[1–100], respectively, and is estimated to be *ρ*~0.63. Furthermore, the PL peak energy increases as the polarization angle *θ* changes from 0° to 90° i.e. from E || [1–100] to E⊥[1–100]. This can be understood by considering the electronic band structure in WZ GaN which has three closely spaced top valence bands (VBs) at the Brillouin-zone center (BZC): (a) heavy hole (HH) [Γ_9_], (b) Light hole (LH) [upper Γ_7_] and (c) crystal field split-off hole (CH) [lower Γ_7_]. For E || [1–100], the transition dominantly involves the HH and LH band whereas the transition involves dominantly the CH band for E⊥[1–100]^39^. Therefore, the PL peak energy for E⊥[1–100] (θ = 90°) is higher than that for E || [1–100] (θ = 0°) as shown in Fig. 6(c). In addition, the PL intensity for E⊥[1–100] (θ = 90°) is lower because of the smaller thermal distribution of carriers for the CH band^40^. Furthermore, the SFs, strain and layer tilt could also have contribution to the polarization properties of (11–22) GaN layer^41^. The detailed optical spectroscopy in the semi-polar (11–22) GaN layers is under investigation by time-resolved photoluminescence and temperature dependent photoluminescence and will be published elsewhere.

## Conclusions

The semi-polar GaN layer (11–22) orientation was grown on *m*-plane sapphire using double AlN buffer layers. The structural and optical properties of semi-polar (11–22) GaN were investigated by XRD, SEM, AFM, TEM, PL. SEM and AFM measurements revealed the GaN surface exhibited the “facet-like” feature with 9 nm of RMS roughness over 5 × 5 μm^2^ surface. The structural properties of the semi-polar GaN layer were found to exhibit strong in-plane anisotropy. The rocking curves of (11–22) GaN reflection are anisotropically broadened with 0.22° FWHM along [-1-123] GaN and 0.42° FWHM along [1–100] GaN. With double AlN buffer layers, the FWHM of XRCs for all the off-axis diffraction decreased significantly, which indicates that the two AlN buffer layers could reduce BSFs and PDs and/or perfect dislocations. The TEM observation showed that the predominant defects in the semi-polar GaN layer were the mixed dislocations. Furthermore, the polarization of the photoluminescence of (11–22) GaN demonstrated the strong emission with the polarization along the [1–100] direction (polarization degree ~ 0.63). The realization of a high polarization semi-polar (11–22) GaN showed the effectiveness of GaN orientation control using double AlN buffer layers and the promising to achieve III-nitride based lighting emission device for displays and backlighting in future.

## Methods

Semi-polar (11–22) GaN epilayers were grown on *m*-plane sapphire substrates using a low pressure metal-organic chemical vapor deposition (MOCVD) system. Trimethylgallium (TMGa), trimethylaluminium (TMAl) and ammonia (NH_3_) were used as the gallium, aluminum and nitrogen sources, respectively. To improve the crystal quality and surface morphology of semi-polar (11–22) GaN layers, we introduced double AlN buffer layers, which consisted of a 20 nm thick low temperature AlN buffer grown at 600 °C, a 60 nm thick high temperature AlN buffer layer grown at 1300 °C. Prior to the growth, the substrate surface was treated in hydrogen atmosphere around 1100 °C and 50 Torr for 20 min. Then double AlN buffer layers were deposited at different temperatures and a reactor pressure of 50 Torr. During the AlN deposition, the flow rates of TMAl and NH_3_ are 110 μmol/min and 2.5 SLM (standard liter per minute), respectively. The V/III source ratio is about 1000. After that, 2~3 μm thick GaN layer was grown at 50 Torrs and 1050 °C. The flow rates of TMGa and NH_3_ are 45 μmol/min and 2.5 SLM, respectively.

The structural properties of GaN samples were examined by high resolution x-ray diffraction (HRXRD: Diffuse X-ray Scattering Station of Beijing Synchrotron Radiation Facility), a Huber five-circle diffractometer was used. The radiation energy of the x-ray beam was 8.05 keV with 0.7 × 0.4 mm^2^ (H × V) of the spot size. The surface morphology of the semi-polar (11–22) GaN layer was analyzed by scanning electron microscopy (SEM) and atomic force microscopy (AFM). The structure of GaN/AlN/Sapphire was studied by conventional transmission electron microscopy (TEM: FEI Tecnai G2 F20), high-resolution TEM (HRTEM) and selective area electron diffraction (SAED), whereas the optical qualities were characterized by room temperature photoluminescence (PL) with a 325 nm line of HeCd laser and the excitation power is 1.0 kW/cm^2^.

## Additional Information

**How to cite this article**: Zhao, G. *et al.* Anisotropic structural and optical properties of semi-polar (11–22) GaN grown on *m*-plane sapphire using double AlN buffer layers. *Sci. Rep.* **6**, 20787; doi: 10.1038/srep20787 (2016).
